# Vapor-Assisted Method to Deposit Compact (CH_3_NH_3_)_3_Bi_2_I_9_ Thin Films for Bismuth-Based Planar Perovskite Solar Cells

**DOI:** 10.3390/mi16020218

**Published:** 2025-02-14

**Authors:** Zihao Gao, Xinjie Wang, Zhen Sun, Ping Song, Xiyuan Feng, Zhixin Jin

**Affiliations:** 1 State Key Laboratory of Metastable Materials Science and Technology, Yanshan University, Qinhuangdao 066004, China; 2 School of Science, Yanshan University, Qinhuangdao 066004, China; 3 School of Microelectronics, Northwestern Polytechnical University, Xi’an 710129, China

**Keywords:** (CH_3_NH_3_)_3_Bi_2_I_9_ thin films, vapor-assisted method, seed layer

## Abstract

Bismuth-based perovskite derivatives, (CH_3_NH_3_)_3_Bi_2_I_9_ (MBI), are promising non-toxic light-absorbing materials widely used in various photoelectric devices because of their excellent stability. However, MBI-based perovskite solar cells (PSCs) are limited by poor film quality, and the performance of such a device is far behind that of lead-based PSCs. In this work, the crystal structure and morphological properties of MBI films were compared across different preparation methods. The two-step vapor-assisted method can prepare continuous dense MBI films because MBI crystal nucleation is induced by the BiI_3_ seed layer. The MBI film grown by this method is better for the production of excellent PSCs compared to the film prepared by the solution method. The best photovoltaic device based on the MBI film could obtain a power conversion efficiency of 1.13%. An MBI device is stored in the glove box for 60 days, and the device’s performance is maintained at 99%. These results indicate that the vapor-assisted deposition of MBI films can be an effective method to improve the performance of bismuth-based planar PSCs.

## 1. Introduction

Lead-based perovskite solar cells (PSCs) have achieved a great breakthrough in power conversion efficiency (PCE), and the certified efficiency currently exceeds 26% [1]. However, the natural defects of lead-based perovskites, such as those related to stability and toxicity, are still the obstacles restricting their industrialization. As a result, several attempts have been made to develop lead-free perovskite materials for photovoltaic devices. For instance, multiple environmentally benign cations, including Sn^2+^ and Bi^3+^, have been explored as replacements for Pb [2,3,4,5]. To date, the PCE of Sn-based PSCs has exceeded 15% [6]. Nevertheless, Sn-based perovskite films face challenges due to the accelerated oxidation of Sn^2+^ to Sn^4+^, which compromises both the device efficiency and long-term stability [2].

Bismuth-based perovskite derivatives have emerged as promising alternatives to lead-based perovskite, owing to their outstanding photoelectric properties and remarkable stability, which position them as ideal candidates for photovoltaic applications [7,8]. Among them, MBI has been subjected to a lot of research work in solar cells due to its excellent stability and simple preparation process, although its PCE remains significantly lower [9,10,11]. At present, the maximum efficiency of MBI solar cells based on mesoporous structures is 3.17%, which is still far behind that of lead and tin-based PSC [12]. One of the main reasons for the low PCE is that the solution fabrication route cannot prepare highly compact MBI films [7,12]. At present, the common solutions of spin coating, two-step soaking and solvent evaporation have not effectively solved this problem [9,13,14,15,16,17,18,19,20,21]. The primary cause is likely due to the significantly faster crystal growth rate of MBI, along with a broader range of solvents dissolving both BiI_3_ and MBI compared to their lead-based counterparts [22].

Past studies have demonstrated that the crystallographic structure and morphology of MBI films are essential for the performance of MBI PSCs [10,23]. However, few studies have been focused on the relationship between the MBI growth method and the film morphology. In this work, MBI films were prepared by various methods, including the spin coating method (SC), two-step soaking method (TSS) and two-step vapor-assisted (TSVA) method. Using BiI_3_ as the seed layer, the crystal growth direction of MBI can be changed by the TSVA method. It was important that the morphology of MBI films was changed from a hexagonal lamellar structure to a continuous dense film. Compared with the traditional SC method, the PCE of Bi-based planar PSCs prepared by the TSVA method could be increased by 4 times, reaching 1.13%. In addition, at room temperature, the PSC retained 99% of its initial performance when stored in a nitrogen atmosphere for two months.

## 2. Results

**Figure 1:**
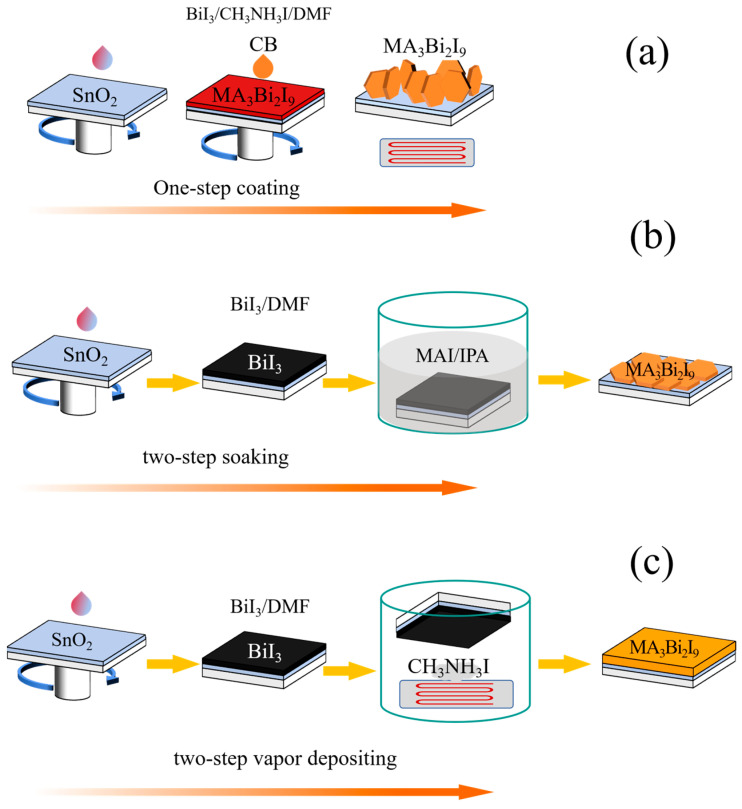
(**a**) Preparation procedure of the MBI thin film by the SC method. (**b**) TSS method. (**c**) TSVA method.

Figure 1 illustrates the preparation of thin films for MBI by different methods. Under the SC method, BiI_3_ and MAI were dissolved in DMF, MBI films were spin-coated on the SnO_2_ substrate, and finally the whole was annealed and crystallized. Under the TSS method, the BiI_3_ film is first spun onto a conductive substrate and then annealed at different temperatures in rapid sequence. Afterwards, the BiI_3_ films are immersed in MAI solution for a certain time and then annealed to form MBI films. At last, the preparation process of the BiI_3_ film by the TSVA method is the same as that under the TSS method. The BiI_3_ was put in a petri dish, which was placed on a hot table in reverse; the BiI_3_ film was facing down, the hot table was placed on the MAI, and the hot table was heated to a certain temperature, after which MBI films were obtained by vapor deposition. For further details on the film fabrication process, please refer to the Supplementary Section.

The morphology of the smooth, dense, and large grains in perovskite solar cells (PSCs) plays a significant role in enhancing power conversion efficiency (PCE) through several interconnected mechanisms. Firstly, large grains reduce the number of grain boundaries and defects, which are known to be sites for carrier recombination. This reduction in recombination losses allows for more efficient charge transport and collection. Additionally, the larger grain size facilitates improved carrier mobility by minimizing scattering events, thereby enhancing the overall electrical conductivity. Furthermore, a smooth surface morphology contributes to superior light absorption by reducing reflection, thereby increasing the number of photons absorbed and converted into charge carriers. Moreover, films with larger grains typically exhibit better crystallinity, which not only improves the mobility of charge carriers but also enhances the stability of the perovskite material, reducing performance degradation over time. Lastly, the reduction in the number of grain boundaries in larger grains minimizes the impact of ionic migration, which is more pronounced in smaller-grained films and can negatively affect both device stability and efficiency [10,13]. Collectively, these factors underscore the importance of a smooth, dense, and large-grain morphology in optimizing the PCE of PSCs.

**Figure 2:**
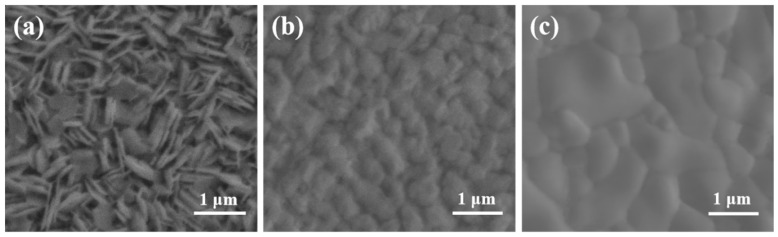
The surface morphologies of MBI films were prepared by different methods. (**a**) SC method, (**b**) TSS, (**c**) TSVA method.

To gain insights into the relationship between film morphology and preparation method, scanning electron microscopy (SEM) was applied. Significant differences in the morphologies of MBI films prepared using different methods were observed (Figure 2). Photographic images of the prepared samples are shown in Figure S1. The MBI polycrystalline film prepared by the conventional SC method exhibited poor morphology, characterized by large grains and noticeable gaps (Figure 2a). The edges of the MBI sheets reached lengths greater than 500 nm. The layer was generally perpendicular to the substrate, so the thickness of the MBI film was about 800 nm (Figure S2). With a change in the MBI growth method, the morphology of the MBI changed accordingly. Figure 2b shows the TSS method, whereby a granular film with small grain sizes was produced, but the film was irregular. Fortunately, the morphology of MBI films prepared by TSVA can be significantly changed. No isolated hexagonal, cross-ridged crystalline grains were noticed in the TSVA-based MBI films. Similar to that observed in lead-based perovskite thin films, the MBI films have a smooth and dense large grain morphology. The grain sizes of the samples were in the range of 500 to 800 nm, with larger grains, exceeding 1 μm, being readily fabricated. Our experiments revealed that the films prepared by the two-step method were more uniform and flat than those prepared by the one-step method. The TSS-based and TSVA-based films showed particle packed structures, while those prepared by the one-step SC method exhibited a vertical hexagonal lamellar structure, consistent with the findings reported in the literature [18,24]. This may be because the two-step method grew the structure on the basis of BiI_3_ as the crystal seed layer, which could regulate the crystal growth direction. In order to verify the causes of the changes in the morphology of MBI films, MBI films were prepared by a two-step spin coating method, which consisted of spin coating BiI_3_ film and MAI films successively, and SEM images were displayed in Figure S3. The film prepared by this method can also form a uniform small granular film. Therefore, the reason for the change in morphology is that, in the two-step preparation process, the BiI_3_ in the first step is used as the crystal seed layer, which can regulate the crystal growth direction during the formation of the MBI film [14,23].

The BiI_3_ films were annealed at 60 °C and 100 °C, respectively. Figure S4a shows the X-ray diffraction (XRD) of BiI_3_ films before and after annealing. The peaks of 12°, 24.5°, 27°, 41°, and 43.6° matched with the crystal faces of (003), (021), (113), (300), and (303) of BiI_3_, respectively [23,25]. The peaks marked with an asterisk (*) in the figure correspond to the diffraction peaks of the FTO substrate. We found that with the increase in temperature, the (300) crystal plane gradually strengthened, and the (003) crystal plane gradually weakened. This indicates that BiI_3_ changes from longitudinal to transverse growth. In the two-step process, the crystal orientation of the seed layer in the first step is a critical factor in the quality of the final product. Hence, BiI_3_’s crystal orientation is the root cause of the vertical or horizontal growth of MBI films [5,23]. Figure S4b presents the ultraviolet–visible (UV-vis) absorption curve of the BiI_3_ films. The BiI_3_ film exhibited a wide absorption in the visible range of 350–680 nm, agreeing well with the reported result [26]. Compared with the unannealed film, the absorption edge of the annealed sample was redshifted, which was caused by the crystallization of the intermediate product (Bi(DMF)_8_–Bi_3_I_12_) prepared by the solution method [27]. When the annealing temperature reached 100 °C, the absorption was obviously enhanced, indicating that the crystallinity of BiI_3_ films was greatly improved. Figure S4c–e present SEM images of BiI_3_ films treated at different annealing temperatures. As the annealing temperature increased, the grain dimension of the BiI_3_ films grew steadily, and a large number of cavities were created on the surface. With the evaporation of DMF from the film, precursor molecules aggregated with each other to form groups, resulting in depletion regions during film growth. The formation of these depleted regions contributed to the development of cavities on the surface of the film [27]. The multihole structure of the BiI_3_ film significantly enhanced the surface area, thereby promoting an effective interfacial reaction with MAI vapor [28].

**Figure 3:**
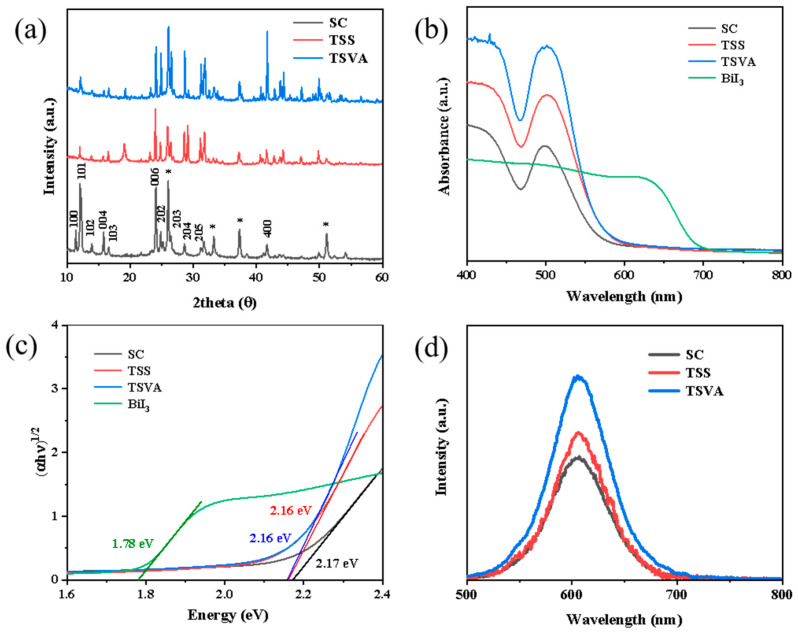
(**a**) XRD patterns. The “*” symbols represent the signals of the FTO substrates. (**b**) UV–visible spectra, (**c**) Tauc plot versus the light energy, (**d**) photoluminescence spectra of MBI films grown via different methods.

The XRD patterns of MBI films grown via different methods are shown in Figure 3a. The main peaks at 2θ = 12.2°, 25.3°and 42.2° correspond to the (101), (006) and (400) crystal faces of the MBI crystal, respectively [7]. By changing the preparation method, the crystal orientations of MBI films changed from a single orientation of layered films to a multi-crystalline orientation of compact flat films. The preponderant crystal direction of the MBI film prepared by the SC method was (101), with (006) and (400) crystallographic directions, in which 006 was the dominant crystal direction. Hence, a vertical layered structure was formed, which also corresponded to the previous SEM. When the preparation methods were changed to TSS and TSVA, the crystal orientation changed obviously. In particular, the crystal orientations of TSVA, MBI included (100), (101), (006), (203), (204), and (400) planes, with no obvious preferred orientation. Therefore, nucleation sites of the BiI_3_ seed layer can grow synchronously in all directions [14]. Therefore, homogeneous and dense MBI films can be obtained by adjusting the crystallization direction. Meanwhile, no residue of BiI_3_ was observed in the film produced by the two-step method, indicating that BiI_3_ was completely transformed into MBI.

Figure 3b shows the UV-vis results of several films. The BiI_3_ film exhibited broad absorption across the visible wavelength range of 350–700 nm, consistent with previous observations. Upon transformation to MBI, the spectrum of the MBI film displayed a distinct exciton absorption peak at 505 nm, which was attributed to the electron transition of Bi^3+^ in the isolated Bi_2_I_9_^3−^ cluster from the ground S_0_ state to the excited 3P_1_ state as the energy of light increased [29]. The MBI films prepared by TSS and TSVA showed significantly higher absorption than the films prepared by SC. The absence of a characteristic BiI_3_ absorption band in the MBI film suggests the complete conversion of BiI_3_. Figure 3c shows the band gap diagram, indicating that the preparation method has no effect on the optical band gap of the MBI film, and the band gap of the prepared film is 2.13 eV. This is basically consistent with the previous values in the literature [23,30]. A Tauc plot was employed to analyze the linear absorption edge by plotting (αhν)^1^/^2^ as a function of hν, revealing a band gap of 1.8 eV for the evaporated BiI_3_ film, consistent with the reported value [31,32].

Photoluminescence (PL) measurements were conducted to further investigate the optical properties of the MBI films. Figure 3d demonstrates the PL spectra of the MBI grown via different methods. The strongest peak at 605 nm (2.06 eV) belonged to the band edge exciton radiation luminescence [22]. The PL intensity of MBI films grown by two-step method is obviously higher than that of MBI films grown by the one-step method. This is attributed to the enhanced crystallinity and morphology of the MBI perovskite film, which suppressed radiative recombination. The strength of the films prepared by the two-step method is better than that of those prepared by the one-step method, which indicates that the crystal quality of bismuth-based perovskite can be improved by the two-step method. The film prepared by TSVA showed the highest PL intensity in these methods, indicating that the film grown by this method had the highest crystal quality. The PL attenuation spectra of MBI films obtained by different methods are shown in Figure S5. The average lifetimes without a charge transport layer fitted via a single-exponential function were 5.83, 6.78 and 7.52 ns for MBI (SC), MBI (TSS), and MBI (TSVA), respectively. The increased lifetime indicates that carriers have a longer diffusion length. Therefore, the photovoltaic device prepared by the two-step MBI film is expected to improve FF and PCE.

**Figure 4:**
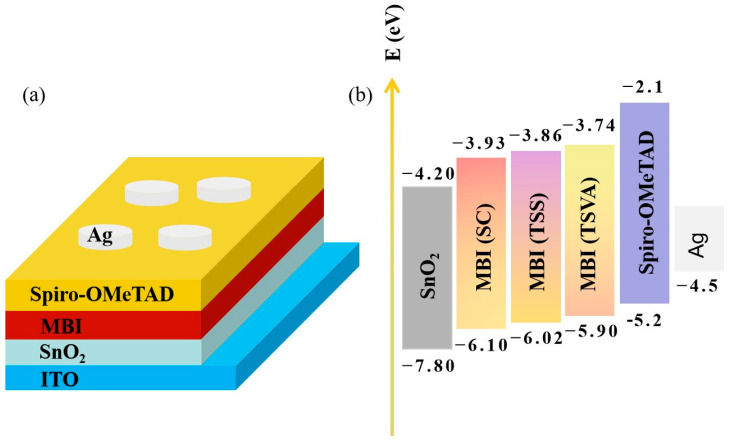
(**a**) Configuration of the planar MBI photovoltaic device; (**b**) schematic band diagram of MBI film grown via different methods.

**Figure 5:**
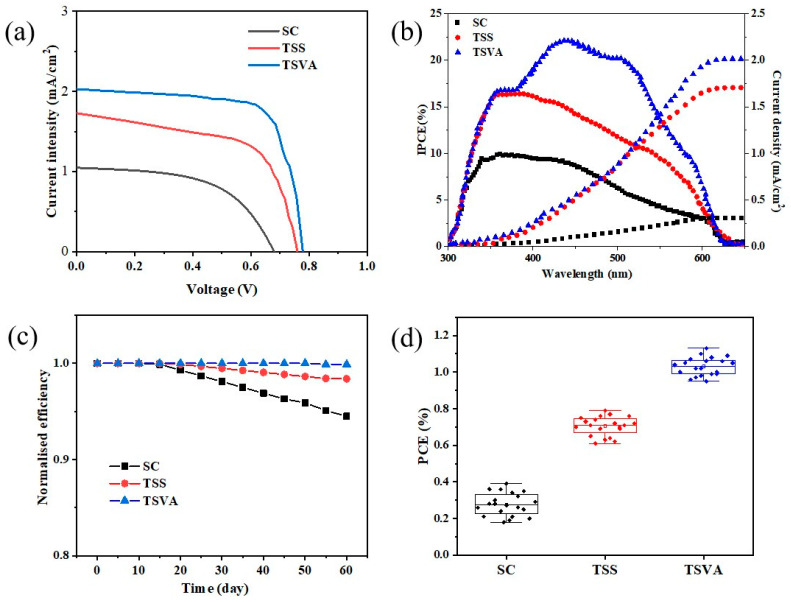
(**a**) *J*-*V* curves of MBI devices with different growth methods. (**b**) IPCE spectra and the integrated current for the different devices. (**c**) Stability of MBI devices with different preparation methods in a glove box. (**d**) PCE box statistics of 20 photovoltaic devices prepared by different methods.

MBI-based PSCs were fabricated using compact films, with the configuration FTO/SnO_2_/MBI/Spiro-OMeTAD/Au (Figure 4a). Figure 4b showed the band locations of all functional layers of the device. UPS measurements (Figure S6) indicate a Fermi level positioned 0.6 eV above the valence band of MBI, highlighting its intrinsic semiconducting nature, which helps reduce carrier recombination. The observation of low radiative recombination in the higher-energy region with a bandgap of more than 2.13 eV was crucial to achieving high open-circuit voltages (*V*_oc_) and efficient photovoltaic conversion. The improved band arrangement of MBI films is anticipated to enhance the collection and transport of charge carriers at the MBI/Spiro-OMeTAD interface, leading to enhanced photoelectric performance [33,34]. The performances of the MBI PSCs are displayed in Figure 5 and Table 1. The best TSVA-based device has demonstrated significant improvement over the other groups in three *J*-*V* parameters. The best device could achieve a short-circuit current (*J*_sc_) of 2.03 mA/cm^2^ while maintaining a high *V*_oc_ of 0.78 V. Additionally, the fill factor (FF) was substantially elevated, contributing to the overall performance enhancement. It is found that the FF of the TSVA-based device exceeded 0.6 in terms of device performance. The enhanced FF is related to the optimization of the crystallinity and morphology of the MBI film, and the improved MBI layer can play a more effective role, given the presence of a charge transport layer. Therefore, the high-quality MBI film prepared by the TSVA route can overcome the bottleneck of high FFs. Figure S7 shows that all PSCs display a small hysteresis behavior.

In accordance with the optical absorption of MBI films prepared using different growth methods, the incident photon-to-current conversion efficiency (IPCE) showed a good correlation. The IPCE spectrum exhibited a maximum value of approximately 23% at 458 nm, and the corresponding integrated current was consistent with that of the *J*-*V* curve. The IPCE curve had a cutoff point at 650 nm and did not have a high platform across the entire visible range. The IPCE of the film prepared by vapor deposition was 2 times higher than that fabricated with the one-step method, and 10% higher than that prepared by the immersion method, which may be due to the higher quality of the film prepared by the vapor deposition method.

In addition to the toxicity of perovskite materials, long-term stability is another key factor limiting the industrialization of PSCs. Bismuth, as a non-toxic element, offers better chemical stability in MBI compared to Pb-based perovskites. The non-encapsulated devices were evaluated over a period of 60 days, showing only a minimal decrease in PCE of 0.1%, demonstrating a significant improvement in stability compared to lead-based perovskites. The long-term stability and its performance over time are depicted in Figure 5c. In addition, the best device was stored in the glove box for 60 days with virtually no attenuation in device performance, maintaining 99.9%. To characterize the reproducibility of MBI PSCs, Figure 5d exhibits the statistical distribution of PCE for the three kinds of MBI PSCs. The photovoltaic device-based MBI grown by TSVA showed a higher average PCE compared to that of solar cells constructed via other methods. Compared with the PSC prepared by the SC method, MBI prepared by the TSVA method showed a narrower PCE distribution range and better reproducibility.

**Figure 6:**
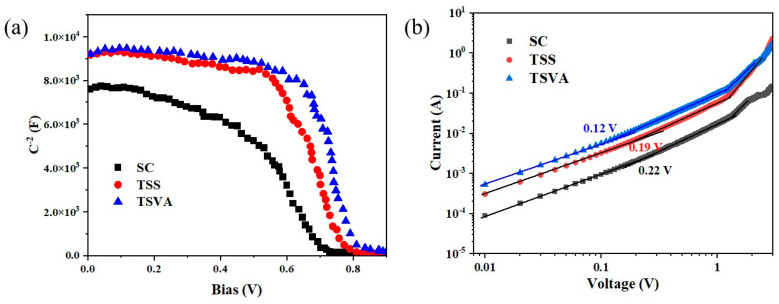
(**a**) C-V curves of different devices yielded by Mott–Schottky testing; (**b**) SCLC curves for hole-only devices prepared via different methods.

The electrical properties of the devices were characterized by Mott–Schottky testing. Figure 6a presents the capacitance–voltage (C-V) characteristics of all devices. The built-in voltages (*V*_bi_) of the SC, TSS and TSVA solar cells showed values of 0.69 V, 0.75 V, and 0.78 V, respectively. The larger *V*_bi_ indicates that the driving force of photogenerated carrier separation increased, resulting in the inhibition of electron–hole recombination [34]. Regarding the MBI films, the large *V*_bi_ could be attributed to the wide bandgap. In addition, in order to contrast the charge transport properties of the devices, the charge space limited current (SCLC) method can be used to study the trap density of the films. Figure 6b shows the SCLC curves of three hole-only devices with MBI films. First, the SCLC curve indicates the Ohmic region at low bias voltage. When the bias voltage surpasses the trap filled limit voltage (*V_TFL_*), the SCLC curve enters the trap-filled region, indicating that the trap state of the device was completely filled. When the bias voltage continued to increase, the device entered the Child’s region [35,36,37]. The trap state densities in the MBI films were calculated using the following equation [38]:

$$N_{t}=\frac{2\varepsilon_{0}\varepsilon_{r}V_{TFL}}{qL^{2}}$$

where *q* is the elementary charge; *L* denotes the thickness of the MBI layer; *ε*_0_ and *ε_r_* represent the vacuum dielectric constant and relative dielectric constant, respectively. When MBI was grown using the TSVA method, the *V_TFL_* of hole-only devices was intended to be 0.12 V, which is lower than that of the MBI films prepared by the SC and TSS methods. This indicates that the trap density in MBI films prepared by this method was significantly reduced. Hence, the trap densities of the TSVA MBI films were calculated to be 1.65 × 10^15^ cm^−3^.

The charge mobility (*μ*) was extracted from the Child region using the Mott–Gurney equation [16]:

$$\mu =\frac{8JL^{3}}{9\varepsilon_{0}\varepsilon_{r}V^{2}}$$

where *J*/*V*^2^ denotes the slope of *J* as a function of *V*^2^ in the Child region. The TSVA MBI film exhibited a charge mobility of 8.5 × 10^−7^ cm^2^V^−1^s^−1^. The trap density and mobility values of MBI films prepared by other methods are shown in Table S2. The results of low trap state density and large charge mobility fully account for the improved *J*_sc_ and *V*_oc_ in MBI solar cells prepared by the TSVA method.

## 3. Conclusions

In summary, different methods (SC, TSS, TSVA) have been applied to prepare the MBI thin films, among which the SC method can produce a hexagonal sheet morphology, while the TSVA method can obtain a uniform and dense surface morphology. The experimental data show that the BiI_3_ seed layer plays a key role in controlling the final morphology and crystallinity of MBI. By controlling the annealing temperature, the crystal direction of the BiI_3_ seed layer changes from 003 to 300, which is conductive to the growth of flat films. During the conversion of BiI_3_ to MBI, the TSVA method can inhibit the preferred orientation of MBI and make the seed layer nucleate uniformly. Further, the preparation of photovoltaic devices with dense MBI as the absorption layer can achieve better performance due to their lower carrier defect density and higher carrier mobility. As a result, the TSVA device has a PCE of 1.13%, and after 2 months of storage in a nitrogen atmosphere at room temperature, the PCE remains at 99% of the initial PCE. Hence, the TSVA method may provide a new way to prepare high-quality MBI films.

## Figures and Tables

**Table 1:** Photovoltaic performance at 100 W/cm^2^ of MBI devices made via several methods.

Sample	*V*_oc_ (V)	*J*_sc_ (mA/cm^2^)	*FF* (%)	*η* (%)
TSVA	0.78	2.03	71.4	1.13
TSS	0.75	1.73	60.4	0.79
SC	0.68	1.05	55.2	0.39

## Data Availability

Data is contained within the article or supplementary material. The original contributions presented in this study are included in the article/supplementary material. Further inquiries can be directed to the corresponding author.

## Supplementary Materials

The following supporting information can be downloaded at: https://www.mdpi.com/article/10.3390/mi16020218/s1, Figure S1: Phototographic images of the prepared samples; Figure S2: SEM of the surface and cross-section of MBI films prepared by spin-coating; Figure S3: The surface SEM of MBI films was prepared by continuous two-step spin-coating; Figure S4: (a) XRD of BiI_3_ films annealed at different temperatures. The “*” symbols represent the signals of the FTO substrates. (b) UV–visible absorption spectra of the BiI_3_ annealed at different temperatures. (c–f) SEM images of BiI_3_, BiI_3_-60, BiI_3_-100 and BiI_3_-150 films; Figure S5: PL decay spectra of MA3Bi2I9 film prepared by different growth methods; Figure S6: UPS spectra and the secondary electron cutoff for the MBI layers produced by different growth methods; Figure S7: Forward and reverse scans of *J*-*V* curves of Bi-based PSC devices prepared by different growth methods; Table S1: Table of photovoltaic parameters of Bi-based PSCs prepared by different methods; Table S2: The carrier mobility and trap density of different preparation methods.
