# Supplementary material for: Vapor-Assisted Method to Deposit Compact (CH3NH3)3Bi2I9 Thin Films for Bismuth-Based Planar Perovskite Solar Cells
Source: Micromachines (Basel). 2025 Feb 14;16(2):218. doi: 10.3390/mi16020218 (PMC11857830; doi:10.3390/mi16020218)
Supplement: Supplementary file 1 [file micromachines-16-00218-s001.zip › micromachines-3448941-supplementary.pdf]

# Supporting Materials

# Vapor-assisted method to deposit compact $(CH_3NH_3)_3Bi_2I_9$ thin films for bismuth-based planar perovskite solar cells

**Zihao Gao [1,2], Xinjie Wang [1,2], Zhen Sun[1,2], Ping Song[1,2], Xiyuan Feng[3,*] and Zhixin Jin[1,2,*]**

[1] State Key Laboratory of Metastable Materials Science and Technology, Yanshan University, Qinhuangdao 066004, PR china.
[2] School of Science, Yanshan University, Qinhuangdao 066004, PR China
[3] School of Microelectronics, Northwestern Polytechnical University, No. 1 Dongxiang Road, Chang’an District, Xi’an 710129, China

## Experimental section

1. Materials

Methylammonium iodide (MAI, 99.5%), Lithiumbis(trifluoromethanesulphonyl)imide-salt (LiTFSI, 99%), 4-tert-Butylpyridine (tBP, 96%), 2,2',7,7'-Tetrakis (N,N-dipmethoxyphenylamine)-9,9'-spirobifluorene (spiro-OMeTAD) and tin(IV) oxide ($SnO_2$) were purchased from Xi'an Yuri Solar Energy Technology Co., Ltd. Bismuth (III) iodide, ($BiI_3$, 99.99% metals basis) was purchased from Shanghai Aladdin Biochemical Technology Co., Ltd. N, N-dimethylformamide (DMF) and Chlorobenzene (CB) were purchased from Beijing J&K Technology Co., Ltd. Ethanol absolute and isopropyl alcohol were purchased from Tianjin Kemiou Chemical Reagent Co., Ltd. In this work, all the materials were used as received withoutf urther purification, unless otherwise specified.

2. Preparation of substrates

Fluorine-doped tin oxide (FTO)-coated glass was etched with Zn powder and 2 M HCl to gain the perfect transparent electrode patterns. The substrates were cleaned by ultrasonication for 25 min in detergent, deionized water, ethanol, and isopropanol, respectively. For drying, the substrates were placed in an oven for 20 min at 60 °C. Then, the substrates were treated with a UV–Ozone Washer for 20-25 min.

Compact-$SnO_2$ substrates: $SnO_2$ was deposited onto the UV–Ozone-treated FTO by spin-coating at 3000 rpm for 50 s, and the substrate was subsequently annealed on a hot plate at 150 °C for 25 min. The spin-coating formulation was prepared as follows: mixing $SnO_2$ solution in a 1:3 volume ratio with deionized water solution.

3. Deposition of $(CH_3NH_3)_3Bi_2I_9$ (MBI) films

3.1 One-step spin-coated MBI film

The MBI precursor solution was prepared by mixing 0.2384 g of MAI and 0.5897 g of $BiI_3$ in 1 mL of N,N-Dimethylformamide, which was filtered using PTFE syringe

filters (0.45 μm) before use. Then, 70 μL of MBI solution was dispensed onto the substrate and spin-coated at 4,000 rpm for 30 s, followed by sintering at 100 °C for 10 min on a hot-plate. During the spin-coating process, we had to add a drop of CB. A reddish film was achieved after the heat treatment.

3.2 Two-step spin-coated MBI film

A two-step spin coating process was employed for the deposition of MBI onto the mesoporous $SnO_2$ film. First, the solution of $BiI_3$ was prepared by dissolving 472 mg $BiI_3$ powder in 1 mL DMF. This was mixed ultrasonically for 30 min before being filtered by use of a PTFE syringe filter (0.45μm). Inside a nitrogen-filled glove box, 20 μL of the filtered solution was spread over the substrate and spin-cast at 4000 rpm for 20 s. The $BiI_3$ film was dried for 30 min before being annealed at 100 °C for 30 min. Subsequently, 100 μL of MAI solution in isopropanol with various concentrations (10 mg/mL) was dropped on the top of the $BiI_3$ film and kept for 30 s, followed by spin-coating at 4000 rpm for 20 s in a glovebox. The film was allowed to anneal at 100 °C for 60 min to form the $MA_3Bi_2I_9$ film.

3.3 Two-step soaking MBI film

First, a 1 mol $L^{-1}$ $BiI_3$ solution in DMF was spin-coated at 6000 rpm for 40 s on a $SnO_2$ layer, obtaining a $BiI_3$ film that transformed from reddish into black after heating at 100 °C for 10 min. Then, the prepared $BiI_3$ thin film was soaked in a pre-prepared MAI solution in isopropanol at a concentration of 15 mg/ml for 15 minutes. After the reaction, it was annealed at 150 °C for 15 minutes to obtain the MBI thin film. After a while, the film changed to reddish immediately, indicating the formation of the MBI perovskite film.

3.4 Two-step vapor deposition MBI film

The $BiI_3$ (1 mol $L^{-1}$) layer was deposited by spin-coating at 4000 rpm for 40 s in a nitrogen-filled glove box and annealed at 100 °C for 10 min. After that, MAI powders were evenly scattered in a Petri dish; the $BiI_3$ was glued to the lid of the Petri dish, and we then closed the lid, followed by vapor-depositing MAI at 180 °C for different times under a nitrogen atmosphere.

4. Fabrication of MBI based devices

After the deposition of freshly prepared MBI films, hole transport was prepared by use of a spiro-MeOTAD solution produced at 4000 rpm for 30 s (prepared by mixing 72.3 mg spiro-MeOTAD, 17.6 μL acetonitrile solution of Li-TFSI (520 mg/mL), 28.8 μL tBP, and 1 mL CB together). The foils were transferred into a vacuum chamber for the further deposition of Au (100 nm) to complete the device fabrication process. The device area was defined as around 0.1256 $cm^2$ by use of a metal mask, the square of which crossed the FTO electrode.

5. Characterizations and measurements

XRD measurements were performed with an X-ray diffractometer (D/MAX-2400, Rigak-u, Japan) with Cu Kα radiation. The diffraction angle was from 10° to 60° at a scanning speed of 5° per min. The morphologies of MBI films were observed by a scanning electron

microscope (FE-SEM, JSM7100F, JEOL, Japan). The absorption spectra were measured in a UV-vis-NIR Spectrophotometer (Model UV-3600, Shimadzu, Japan) in the range of 500-800 nm. The incident photon-to-electron conversion efficiency (IPCE) was measured on a solar cell measurement system from PV Measurement Inc. Steady-state photoluminescence (PL) spectra were acquired using an FLS980 (Edinburgh Instruments, United Kingdom). The current density–voltage (*J-V*) curves were recorded using a Keithley 2400 source-meter under 1 sun illumination (AM 1.5 G, 100mW $cm^{-2}$), which was calibrated using a standard silicon reference cell. The capacitance–voltage (C-V) was measured using an electronchemical workstation (Princeton PARSTAT 2273) at a voltage from 0 to 0.8 V.

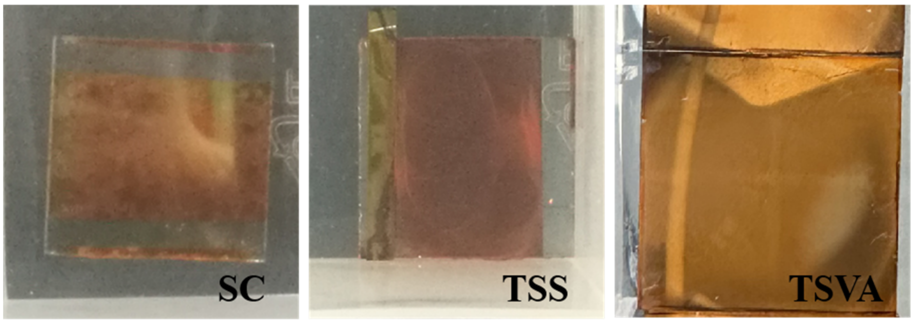

Figure S1. Phototographic images of the prepared samples.

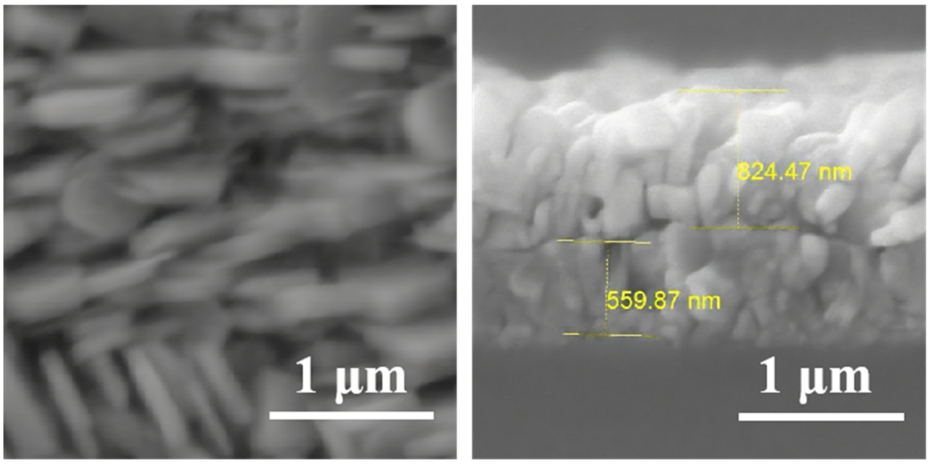

Figure S2. SEM of the surface and cross-section of MBI films prepared by spin-coating.

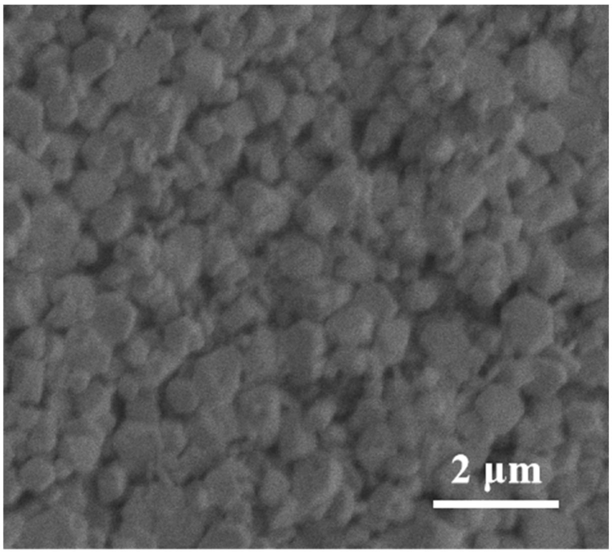

Figure S3. The surface SEM of MBI films was prepared by continuous two-step spin-coating.

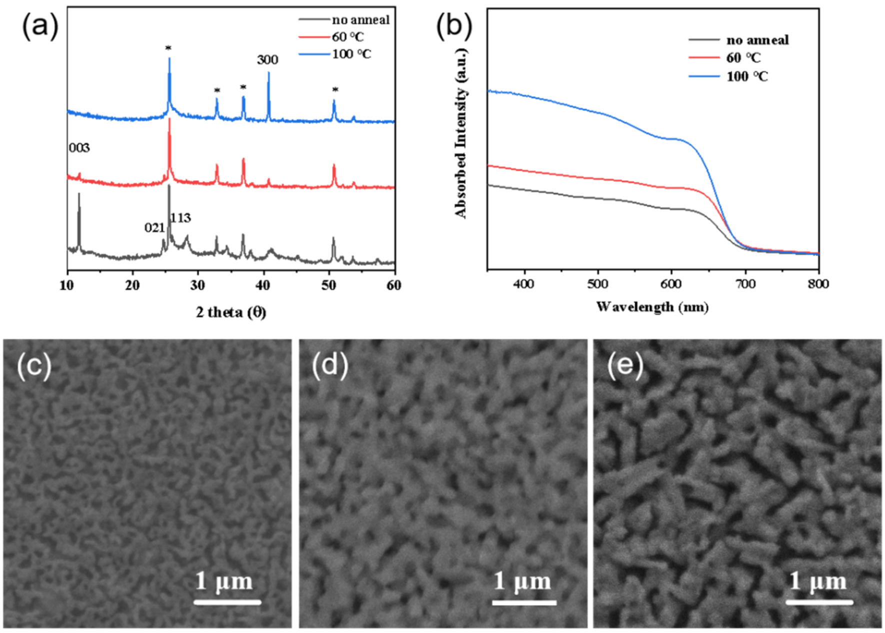


Figure S4. (a) XRD of $BiI_3$ films annealed at different temperatures. The "*" symbols represent the signals of the FTO substrates. (b) UV–visible absorption spectra of the $BiI_3$ annealed at different temperatures. (c-f) SEM images of $BiI_3$, $BiI_3$-60, $BiI_3$-100 and $BiI_3$-150 films.

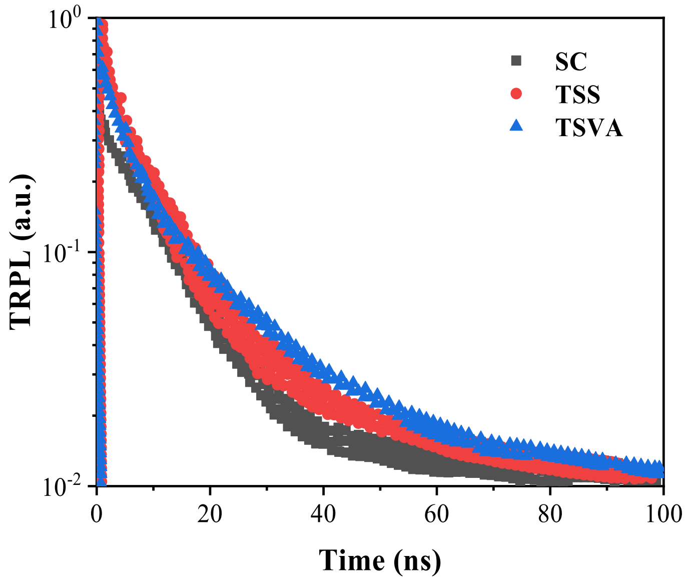


Figure S5. PL decay spectra of $MA_3Bi_2I_9$ film prepared by different growth methods.

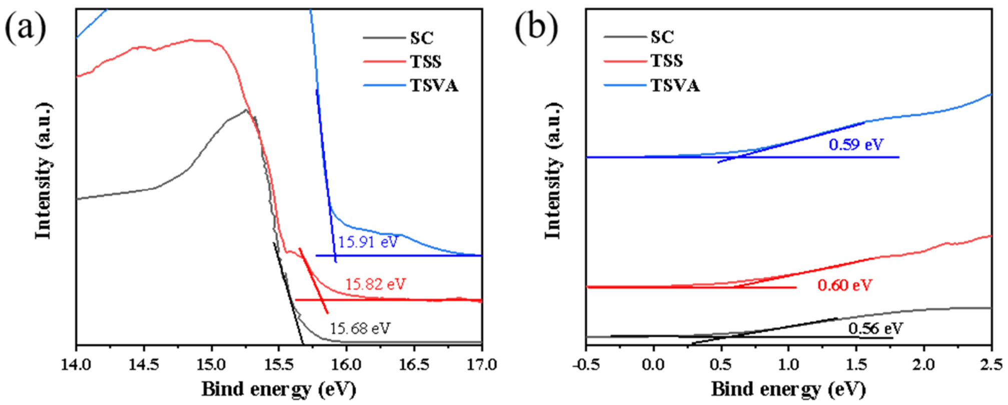


Figure S6. UPS spectra and the secondary electron cutoff for the MBI layers produced by different growth methods.

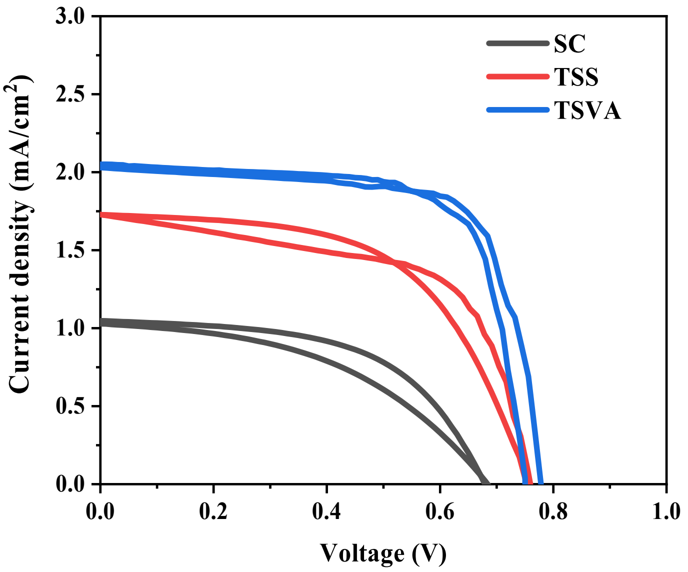


Figure S7. Forward and reverse scans of *J*-*V* curves of Bi-based PSC devices prepared by different growth methods.

Table S1. Table of photovoltaic parameters of Bi-based PSCs prepared by different methods.

| sample | $V_{oc}$ (V) | $J_{sc}$ (mA/cm$^2$) | $FF$ (%) | $\eta$ (%) |
|---|---|---|---|---|
| | 0.78/0.75 | 2.03/2.05 | 71.4/70.2 | 1.13/1.08 |
| | 0.75/0.75 | 1.73/1.73 | 60.4/57.0 | 0.79/0.74 |
| | 0.68/0.68 | 1.05/1.03 | 55.2/45.7 | 0.39/0.32 |

Table S2. The carrier mobility and trap density of different preparation methods.

| sample | carrier mobility | $V_{TFL}$ | trap density |
|---|---|---|---|
| SC | $1.43 \times 10^{-8}$ cm$^2$ V$^{-1}$ s$^{-1}$ | 0.22 V | $4.14 \times 10^{15}$ cm$^{-3}$ |
| TSS | $9.26 \times 10^{-7}$ cm$^2$ V$^{-1}$ s$^{-1}$ | 0.19 V | $2.28 \times 10^{15}$ cm$^{-3}$ |
| VSTA | $8.52 \times 10^{-7}$ cm$^2$ V$^{-1}$ s$^{-1}$ | 0.12 V | $1.65 \times 10^{15}$ cm$^{-3}$ |
